# Design of PD-L1 inhibitors for lung cancer

**DOI:** 10.6026/97320630015139

**Published:** 2019-02-28

**Authors:** Trishang Udhwani, Sourav Mukherjee, Khushboo Sharma, Jajoriya Sweta, Natasha Khandekar, Anuraj Nayarisseri, Sanjeev Kumar Singh

**Affiliations:** 1 In silico Research Laboratory, Eminent Biosciences, Mahalakshmi Nagar,Indore 452010,Madhya Pradesh,India; 2 Bioinformatics Research Laboratory,LeGene Biosciences Pvt Ltd., Mahalakshmi Nagar,Indore 452010,Madhya Pradesh,India; 3 Computer Aided Drug Designing and Molecular Modeling Lab, Department of Bioinformatics, Alagappa University, Karaikudi 630 003, Tamil Nadu, India.

**Keywords:** Lung cancer, PD-L1 inhibitors, Molecular docking, Virtual screening, Cytotoxicity studies, ADMET

## Abstract

The progression of lung cancer is associated with inactivation of programmed cell death protein 1, abbreviated as PD- 1 which regulates the suppression of the body's immune system by suppressing T- cell inflammatory activity and is responsible for preventing cancer cell growth. It is of interest to identify inhibitors for PD-L1 dimeric structure through molecular docking and virtual screening. The virtual screened compound XGIQBUNWFCCMAS-UHFFFAOYSA-N (PubChem CID: 127263272) displays a high affinity with the target protein. ADMET analysis and cytotoxicity studies further add weight to this compound as a potential inhibitor of PD-L1. The established compound BMS-202 still shows the high re-rank score, but the virtual screened drug possesses a better ADMET profile with a higher intestinal absorption value and lower toxicity.

## Background

There are 8.8 million recorded deaths of malignant cancer every year according to WHO data and this number keep on increasing which is a clear indication of the threat this disease poses. Studies in the past decade have confirmed that the immune system displays a variety of mechanisms to combat the growth of cancer cells in the body. Hence, in order for the cancer cells to grow and develop, the cells have to find ways to repress these immunological mechanisms. One such mechanism used is altering the expression of co-inhibitory and co-stimulatory articulated molecules [1]. Cancer immunotherapy is increasingly being used in recent clinical treatments in order to overcome tumorinducedimmunosuppression. Immune checkpoint blocking (ICB) antibodies targeting programmed death protein 1 (PD- 1)/programmed cell death ligand 1 (PD-L1) and cytotoxic-Tlymphocyte- associated protein 4 (CTLA4) clinically prove that treatment is possible through immunity modulation [2]. Programmed cell death protein 1, abbreviated as PD- 1, is a regulatory protein involved in the suppression of the body's immune system by suppressing T- cell inflammatory activity. It is present on the cell surface and helps in the prevention of autoimmune diseases. But this protein can also help the cancer cells by preventing them from getting killed by the immune system. PD- 1 shows an affinity towards two proteins of the B7 family: PD-L1 (B7-H1, CD274) and PD-L2 (B7-DC, CD273). When PD-1 expressed on T cells interacts with its two ligands, its functional activities are reduced, including cytokine secretion, proliferation, and cytolytic activity [3]. This ligand interaction down regulates the T-cell response during the growth of the tumor. Clinical studies of cancer cells have shown elevated levels of PD-L1, which proves that the cancer cells abuse this feature.

Until now, monoclonal antibodies such as Nivolumab and Pembrolizumab (which bind to PD-1) and Avelumab and Durvalumab (which bind to PD- L1) have gained the U.S. Food and Drug Administration acceptance other than several others which are in various phases of trials. The antibodies approved are directed against metastatic non-small-cell lung cancer (NSCLC), melanoma, and renal cell carcinoma. The development of small molecules that are targeted towards the inhibition of this interaction is lagging as compared to the development of monoclonal antibodies. Small molecules for the same resolution could offer many advances that might be complementary and potentially better than large biological molecules [4]. For example, small-molecule drugs can be directed towards intracellular targets which are inaccessible to protein therapeutics. They can be orally administered and gave the potential to reach high exposure levels inside the tumor microenvironment. Moreover, these can be prepared at lower costs as compared to antibodies.

A series of small molecules targeting the PD-1/PD-L1 interaction have been reported [4-6]. Bristol-Myers Squibb (BMS) recently disclosed the first non-peptidic small molecule inhibitors against the PD-1/PD-L1 pathway that highlighted the activity in a homogeneous time-resolved fluorescence (HTRF) binding assay [7-8]. It has been shown that these compounds bind directly to PD- L1, not to PD-1 and dissociate the PD-1/PD-L1 complex in vitro. Further, it has also been confirmed that these molecules inhibit the PD-1/PD-L1 interaction by inducing PD-L1 dimerization through PD-1 interacting surface. Besides BMS series, other peptides and peptido-mimetics have been discovered by Aurigene researchers with interactions in mouse splenocyte proliferation assays, human peripheral blood mononuclear cell (PBMC) proliferation assays, IFNg production in a CTL assay, and inhibition of tumor growth after subcutaneous injection of mouse melanoma cells into mice. Extensions of these studies toward cyclo-peptide inhibitors of the complex formed were detailed in another recent patent from Aurigene with both open-chain and cyclized derivatives [9]. Furthermore, tripeptide peptido mimetics including diacylhydrazine and urea linker moieties and peptides contain the diacylhydrazine and urea linkers with broader variations of the amino acid building blocks have been put forward by Aurigene in another patent [10]. Taking the concept further, the Aurigene synthesized oxadiazole and thiadiazole moieties into the core chain of the peptide backbone [11-12]. Owing to the fact that the development of these small molecule inhibitors is relatively lacking, the present study aims to identify a potential small molecule inhibitor, which binds with PD-L1 with high affinity and can hence be carried for further trials for the clinical treatment of lung cancer. Therefore, it is of interest to identify inhibitors for PDL1 dimeric structure through molecular docking and virtual screening.

## Methodology

### Selection of PD-L1 inhibitors:

**Figure 1:**
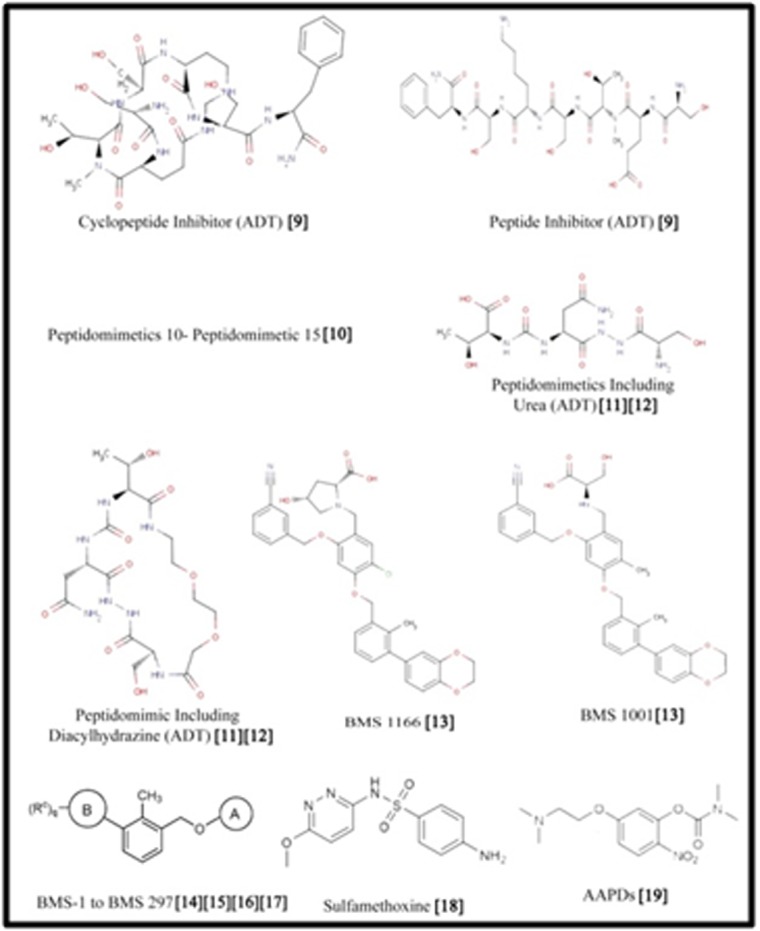
Established Inhibitors of PD- L1 which do not have PubChem ID with references

Literature studies were carried out to search for established inhibitors of PD-L1 ligand, which were capable of binding and hence inhibiting the activity of the protein. The total number of established inhibitors was found to be 311, which were chosen for further analysis. Out of the 311 small molecule inhibitors, only one was found to have a PubChem ID (Table 1) while the structures of others were not available. The 3D structures of all these compounds were constructed using MarvinSketch and were saved in the 3D.sdf format (Figure 1).

### Protein and ligand Preparation:

**Figure 2:**
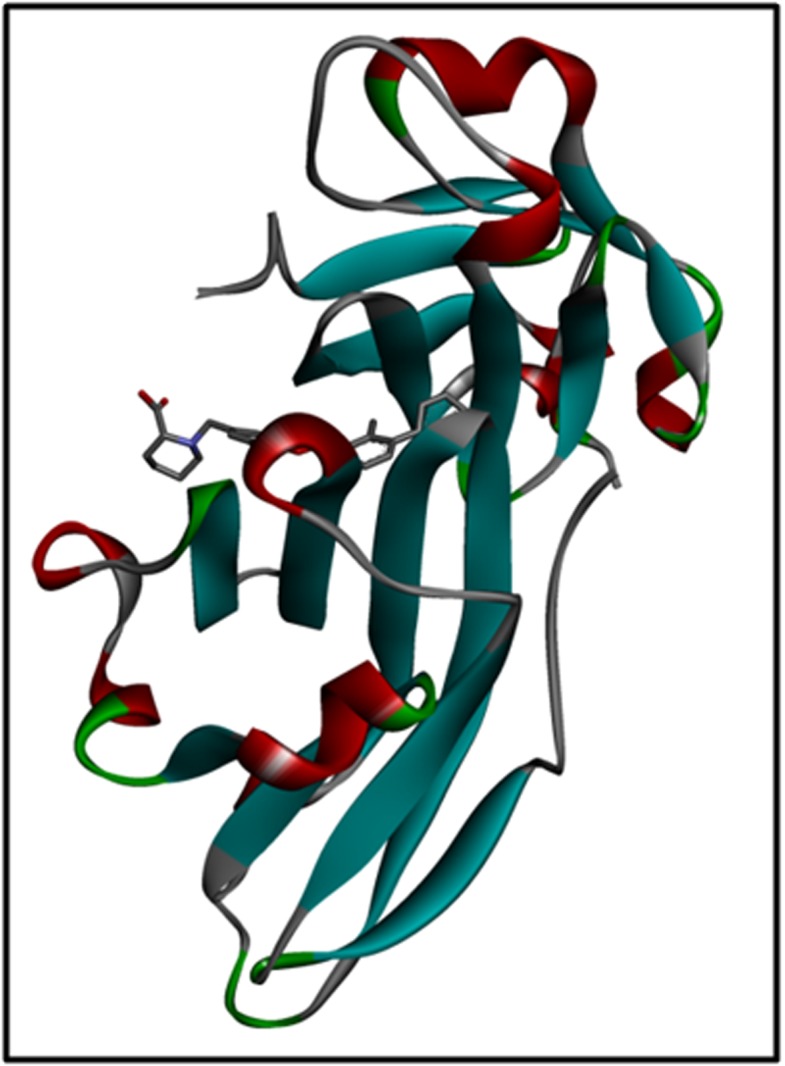
3D structure of PD-L1 dimer (PDB ID: 5J8O) generated using Discovery Studio

The protein 3D structure or the crystal structure of the target protein i.e. the PD-L1 dimer was taken from the Protein Data Bank (PDB) with the PBD ID: 5J8O [20]. This structure was saved for docking (Figure 2). Furthermore, ligand preparation was done by utilizing the 3D structures of all the constructed as well as the retrieved ligands which were embedded in the LigPrep module of Schrodinger suite, 2013 (Schrodinger. LLC, New York, NY) and were optimized with the help of the OPLS 2005 force field algorithm [21-26]. This preparation gave the ligands in a single file, which was saved with a .sdf extension for docking with the protein crystal structure [27-31].

### Molecular docking:

The molecular docking investigations were carried out by using Molegro Virtual Docker (MVD) which unified with high potential Piece-Wise Linear Potential (PLP) and MolDock scoring function [32-35]. The single ligand file prepared in the previous section was used. Protein preparation was carried out by removing the preexisting ligand form the protein structure. Before removing the ligand, the cavity in which the ligand existed was seen. Cavity one was observed to have the largest volume and the presence of the ligand and was hence chosen for docking of the prepared ligands. Docking procedure holding parameter of maximum iteration of 1500, grid solution 0.2 having a binding affinity, maximum population size 50, the protein and ligands were assessed on the subsequent confirmation of the Internal Electrostatic interaction (Internal ES), sp2-sp2 torsions, and internal hydrogen bond interaction [36-42]. The binding site outlined the first cavity according to high volume. A post dock study comprised of energy minimization and H-bond optimization. Placing of Simplex Evolution at max steps 300 and neighbour distance faster 1.00 [43-47]. After docking to minimize the complex energy of ligandreceptor interaction the Nelder Mead Simplex Minimization (using non- grid force field and H-bond directionality) was used [48-51].

### Virtual screening:

The compound with PubChem ID of 117951478 which is BMS- 202 was used to carry out a similarity search to attain a better compound having a greater binding affinity to the dimer structure other than any previously established drugs [52-57]. The similarity searching was carried out against the PubChem database developed by NIH, one of the public chemical repositories which consist of structures of 93 million chemical compounds. The filtrations property parameter set by the component rule of Lipinski's rule of five was set at threshold >=95 [58-64]. A similar similarity searching was carried out against the ZINC database which has especially been designed for virtual screening purposes. These compounds were then docked with the same procedure with the target protein PD-L1 to discover the compound having surpassed binding affinity to the protein.

### Drug-Drug Comparative Study:

The 'unnamed complex' structure file generated in the established drug docking result folder and was opened in Molegro Virtual Docker. All the constraints, cavities and ligands in the structure were removed to obtain only the protein structure [33, 37, 40]. The best pose of the drug was then imported and the structure generated was saved as the best-posed drug and was saved in PDB format. Similarly, the 'unnamed complex' structure file was retrieved from the virtual docking result and the steps were repeated to obtain the best virtual screened drug pose [65-72]. An excel sheet was organized to check and compare all the affinities, hydrogen interaction, steric energy and high re-rank score to draw out a comparison between the two drugs [73-79].

### ADMET studies:

The admetSAR database available at http://lmmd.ecust.edu.cn :8000/ offers an open and free interface to search for the biological and chemical profile of a compound. The properties mentioned in the ADMET profile such as digestion, absorption, metabolism, toxicity, excretion and so on, provide us with essential information related to the development and discovery of drugs. The admetSAR database mostly consists of 22 qualitative classifications and 5 quantitative regression models, which aid in providing the outcome with high precision based on the prediction. Hence the estimation of the properties of the compounds was predicted using admetSAR. The properties of the established compound and virtual screened compound such as the bioactivity properties and toxicity were predicted by using admetSAR [33, 37, 40].

### Software, Suites and web servers used:

The 3D chemical structures were retrieved from NCBI's PubChem database in 3DSDF format. Some compounds that lack PubChem ID or the 3D structure was unavailable in PubChem were drawn with the help of MarvinSketch5.6.0.2, (1998-2011, ©Chem AxonLtd). Schrodinger suite was used for the optimization of ligands (Schrodinger, LLC, 2009, New York, NY). The flexible docking was achievedby taking receptor protein structure and all ligand compounds in Molegro Virtual Docker 2010.4.0.0. Molecular Visualization was done with the assistance of Accelrys Discovery Studio® Visualizer 3.5.0.12158 (© 2005-12, Accelrys Software Inc.). ADMET profiles were obtained and tabulated using admetSAR (Laboratory of Molecular Modeling and Design, ©2012 East China University of Science and Technology, Shanghai Key Laboratory for New Drug-Drug Design). Cytotoxicity study was conducted using CLC-Pred (Way2Drug © 2011 – 2018).

In silico studies require methods of phenotypic screening to decrease the time as well as the cost of the experiments that would be conducted in vivo for the screening of anticancer agents through millions of natural and synthetic chemical inhibitors. Previously established PASS (Prediction of Activity Spectra for Substances) algorithm was used to produce and confirm the classification SAR models for calculating and predicting the cytotoxicity of inhibitors against varying kinds of human cell lines using ChEMBL experimental data. By utilizing the provided SAR models, a freely available web-service was developed for cell-line cytotoxicity profile prediction (CLC-Pred: Cell-Line Cytotoxicity Predictor) based on their structural formula. This webservice resides athttp://way2drug.com/Cell-line/. After the input is provided in the web service, probabilities are given in the form of 'Pa' (probability "to be active") which gives the estimate chance of the input compound fitting into the sub-class of active compounds, and Pi (probability "to be inactive") which gives the estimate chance of the input compound belonging to the sub-class of inactive compounds.

## Result and Discussion:

### Docking results:

The docking results of the pre-established 311 drugs established BMS-202 as the compound showing the best interaction (Table 2). This compound has PubChem CID-117951478 and shows the highest affinity score directed towards our target protein and has properties such as molecular weight of 419.525 g/mol, hydrogen bond donor count of 2 and hydrogen bond acceptor count of5. The logP value is established at 3.6. Hence, this compound discloses greater inhibition over protein PD- L1. Similarity searching for this inhibitor resulted in two similar compounds against PubChem. Table 3 shows the docking result of these two virtual screened compounds. The table shows that the compound SCHEMBL19100243 (PubChem CID- 127263272) has the highest affinity. This compound has a molecular weight of 455.983 g/mol, 3 hydrogen bond donors, and 5 hydrogen bond acceptors. Similarity searching against ZINC database displayed 468 similar compounds with a compound with Zinc ID ZINC22037432 showing the highest affinity with the PD- L1 structure (Table 4). This compound has a molecular weight of 372.493, 2 hydrogen bond donors, 7 hydrogen bond acceptors, and a logP value of 1.13.

### Drug-drug comparative result:

Table 5 gives an account of the MolDock and re-ranks scores of the best-posed drug and the virtual screened drug when docked with the dimeric structure of the PD-L1 protein structure, along with other important parameters. It is noteworthy here that the total energy of BMS-202 inhibitor interacting with the PD-L1 dimeric structure is better when compared to the entire virtual screened compounds with preferable affinity but the difference in the interaction energies is minimum. However, it is surprising to note that interaction scores of the virtual screened drug (CID: 127263272), such as External Ligand interactions as well as proteinligand interactions, lie very close to the corresponding scores of the established drug BMS- 202 (CID: 117951478). A similar observation is made in case of steric interactions by PLP and LJ12-6. The difference observed when comparing these parameters is quite small. However, the established drug does show higher stability based on hydrogen bonds when compared to the virtual screened drug. Based on these observations, it can be said that the virtual screened drug puts forward a strong case in favour of this compound showing as effective inhibitory properties as compared to the established drug, if not better, towards the target protein PDL1.

### Pharmacophore mapping:

**Figure 3:**
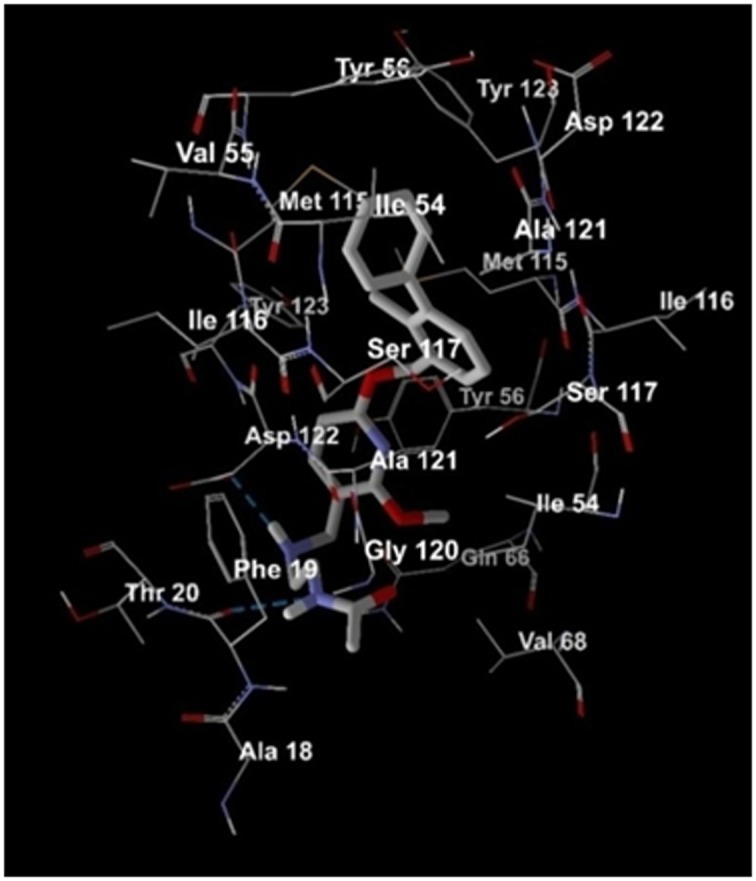
The Virtual Screened Drug CID: 127263272, the most effective drug shows hydrogen bond interactions

Pharmacophore mapping helps to provide necessary spatial systematic topographies of molecular interaction with a particular target receptor other than the technique of molecular docking. Pharmacophore studies deliver an accurate query on the optimum interaction of the drug with its target protein, assisted by annotations and denote the aligned poses of the molecule and aid us to find the high interaction mode between target protein and compound. As the interaction of the receptor PD-L1 and the drug with virtual screened drug PubChem CID-127263272, is found to be quite effective, pharmacophore studies are carried out to study various interactions presented in the complex formed. The various interactions that were mapped consisted of hydrogen bond interactions, van der walls (vdW) interaction, ligand interactions. Figure 3 highlights the hydrogen bond interaction of the preexisting, established compound BMS-202 with PubChem CID: 127263272 outlining high-affinity score interposed the active site of the dimeric structure of PD-L1 receptor protein. Tiny blue dotted lines show hydrogen bond interaction of specific amino acids in the receptor, with the drug when the most stable complex is formed. The figure displays that three amino acids viz. Glycine 120, Phenylaniline 19 and aspartic acid 122 form hydrogen bonds with the virtual screened drug.

**Figure 4:**
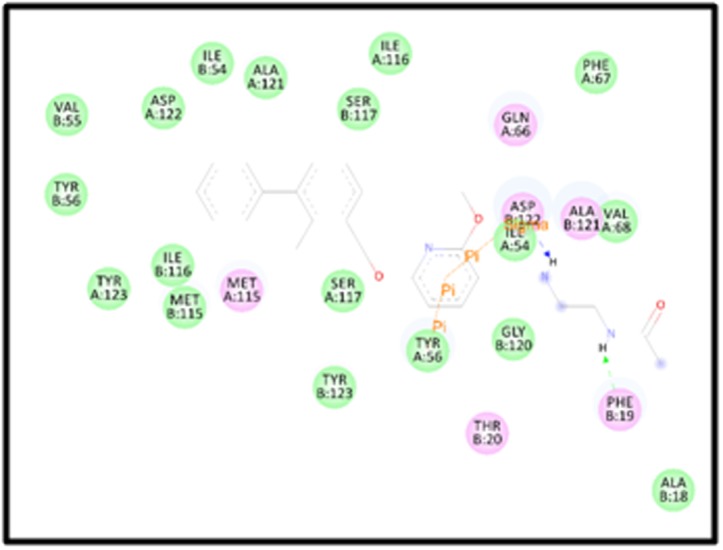
The Virtual Screened Drug CID: 127263272, the most effective shows van der Waals interactions.

**Figure 5:**
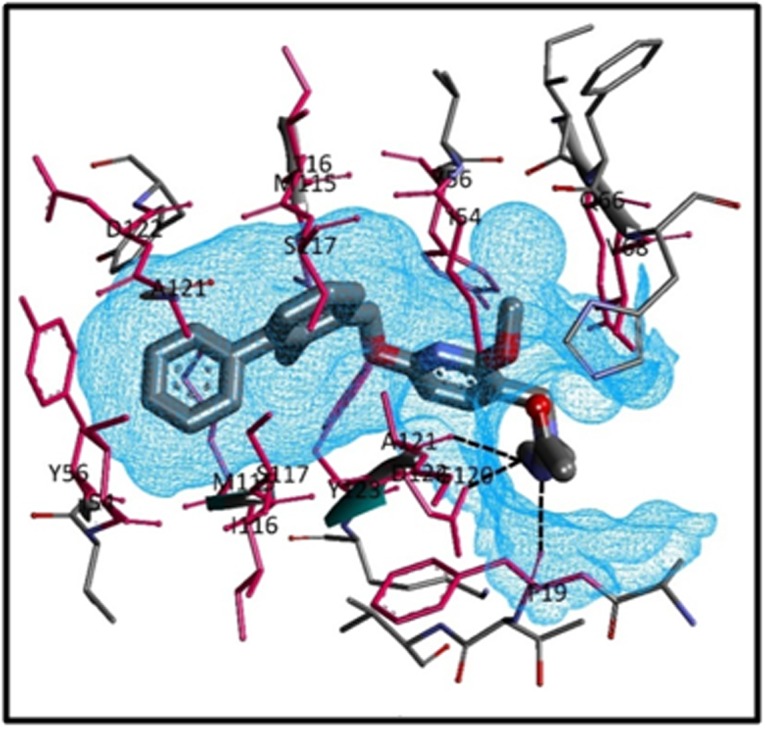
The Virtual Screened Drug CID: 127263272, the most effective drug in the active site of the protein with ligand interactions

The illustration in Figure 4 displays the interaction of the residues with high-affinity drug PubChem CID: 127263272embedded in the receptor. The green circles represent van der Waals interaction and the residues highlighted in the pink circles are the ones that show electrostatic interactions. A green arrow and blue arrows between residues and ligand highlight Hydrogen bond interaction. It can be seen from Figure 4the residues Phe B:19 and Asp B:122 represents the hydrogen bond formation in the complex formed. As shown in the figure in the high- volume cavity of the dimeric structure of PDL1, the inhibitor reveals a green arrow to Phe B: 19 which focuses drug as the hydrogen bond donor. Accordingly, it can be seen that many of the residues show van der Walls interaction with the drug. Val B:55, Asp A:122, Ile B:54, Ala A:121, Ser B: 117, Ile A:116, Phe A:67, Val A:68, Ile A:54, Ala B:18, Gly B:120, Tyr A:56, Tyr B:123, Ser A:117, Met B:115, Ile B:116, Tyr A:123, Tyr B:56 can be seen to have van der Walls interactions. Furthermore, pi-pi interaction between the residue Tyr A:56 and the compound and pi- sigma interaction between Asp B:122 and the compound can be observed. Figure 5 portrays the compound PubChem CID: 127263272 in the active binding site of protein PD-L1 with H-bond interactions. Interactions are indicated by black dotted lines, clearly visible in the figure between the drug and Glycine 120, Phenylaniline 19 and aspartic acid 122 in the protein cavity.

### ADMET profile

Table 6 summarizes the ADMET prediction of both the best-docked compound PD1-PDL1 inhibitor PubChem CID: 117951478 and XGIQBUNWFCCMAS-UHFFFAOYSA-N PubChem CID: 127263272. It can be seen that the ADMET profiles of both these compounds are approximately equivalent in some parameters, while in others the virtual screened compound presents better figures. Observations from the table show that brain penetration prediction that is Blood Brain Barrier (BBB), shows a positive result (+) which indicates that it is positive for absorption. Human Intestinal Absorption (HIA), which is the prediction of absorption of the drug in the intestine, shows a very slightly higher probability in case of the virtual screened drug as compared to the established drug. The Pglycoprotein Substrate and P-glycoprotein Inhibitor predictions of both the compounds display an alternating similar probability. The absorption site for the P-glycoprotein substrate for compound PD1-PDL1 inhibitor reveals a higher probability than XGIQBUNWFCCMAS-UHFFFAOYSA-N. Conversely, P-glycoprotein Inhibitor shows the values with high probability in the case of XGIQBUNWFCCMAS-UHFFFAOYSA-N. In addition to the distribution of subcellular localization in both the compounds are mitochondria. The mitochondrial distribution, both the compounds shows the distribution in very close proximity to each other. Metabolism predictions vary in points like CYP450 3A4, CYP450 2C9 Inhibitor, CYP450 3A4 Inhibitor, CYP450 2C19 Inhibitor, and CYP450 3A4 Inhibitor with both the compounds acting as substrate as well as the inhibitors. Both the compounds display comparable high inhibitory effect towards the target protein. Further study of bioactivity in the profile of toxicity is almost equivalent. Table 7 summarizes the comparison of the regression prediction of ADMET analysis of the two drugs under consideration. The regression model shows that the virtual screened drug has a higher CaCo2 permeability in regression studies. Toxicity studies show almost equivalent values with the model categorized in Rat Acute Toxicity, Fish Toxicity and Tetrahymena Pyriformis Toxicity.

### Comparative ADMET profile study of the compounds and the control

**Figure 6:**
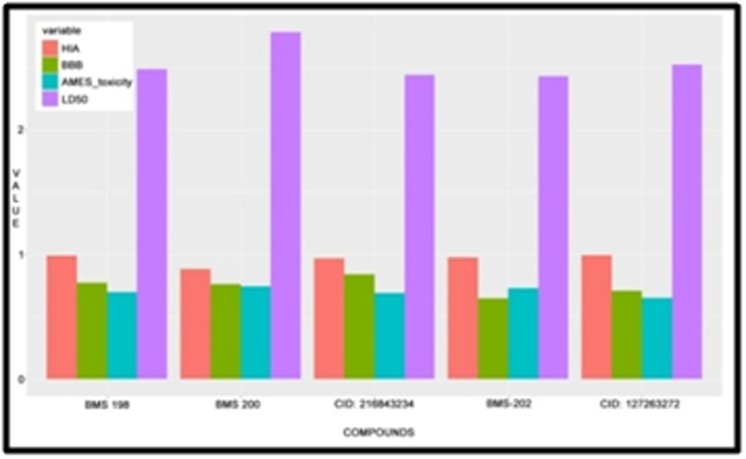
Comparative ADMET studies of BBB, HIA, AMES toxicity and LD50 of the Established compounds against Virtual screened compounds.

A relative ADMET profile comparison was carried out for selected inhibitors. Predictions were based on parameters such as the Blood- Brain Barrier (BBB), Human Intestinal Absorption (HIA), AMES Toxicity, and LD50 rat toxicity. The established inhibitor PD1-PDL1 inhibitor, BMS- 202, the virtual screened drug XGIQBUNWFCCMAS-UHFFFAOYSA-N, CID: 127263272 and three other top compounds BMS- 198, BMS- 200 and PubChem CID 126843234 were taken up for comparison according to ADMET studies. These five inhibitors were graphically represented using Rprogramming as represented in Figure 6. The parameters, BBB, HIA, AMES Toxicity, and LD50 acquired from the admetSAR database and were tabulated according to their estimated values. BMS 200 is the sole inhibitor that displays a negative Blood Brain Barrier (BBB). Also, this inhibitor shows higher LD50 toxicity in rats well as AMES Toxicity when compared to others. The virtual screened compound shows lowest AMES toxicity while the established drug shows the lowest LD50 rat toxicity value (Table 8).

### Cytotoxicity study:

The results of cytotoxicity studies for the best virtual screened compound that is compound with PubChem CID 127263272 are summarized in Table 9. The table provides the probability of the compound to fall in the category of "active" compounds indicated by 'Pa' value. This implies that the structure of this compound bears a resemblance to the structures of molecules that are the most typical in a subset of "actives" in the PASS training set. For small cell lung cancer, the Pa value is 0.256 for the cancerous cell line NCI-H69. The 'Pi' value, which provides the probability of the compound falling in the category of "inactive" compounds, for the same cell line is a lowly 0.07. Interestingly, the same compound gives a high "Pa" value of 0.516 for the cancerous cell line for childhood acute myeloid leukemia with maturation named Kasumi 1, which is isolated from hematopoietic and lymphoid tissue. The 'Pi' value for the same cell line is just 0.035. This result may implicate the usefulness of the same drug in the clinical treatment of acute myeloid leukemia.

## Conclusion

The BMS-202 drug, introduced by the Bristol-Myers Squibb (BMS) still binds to the PD-L1 dimeric structure with the highest affinity. We show that the compound XGIQBUNWFCCMASUHFFFAOYSA- N, CID: 127263272 which, even though displays a slightly lower binding affinity, displays some properties that project it to be at better when compared to BMS- 202. The virtual screened drug displays a better ADMET profile when compared to BMS-202. The pharmacophore interactions of the drug portray that the drug binds to the dimeric PD-L1 structure with three hydrogen bonds. The presence of numerous van del walls (vdW) interactions further adds to the binding affinity of the drug to the protein structure. Cytotoxicity studies confirm that the molecular has a potential of acting as an effective inhibitor pending further in vitro analysis.

## Conflict of Interest

The authors declare no conflict of interest, financial or otherwise.

## Figures and Tables

**Table 1:** Established Inhibitors of PD- L1 with PubChem ID and properties

S. No.	Pub ID	Inhibitor	M.W. (gm/mol)	Molecular formulae	H-Bond donors	H-bond acceptors	LogP	Reference
1	117951478	BMS-202	419.525 g/mol	C25H29N3O3	2	5	3.6	[11] [12]

**Table 2:** Established drug docking result

Ligand	Filename	MolDock Score	Rerank Score	Interaction	HBond
117951478	[00] 11795147	-208.769	-173.766	-226.856	-5.23353
BMS 198	[00] BMS 198	-208.811	-171.797	-223.056	-2.85593
BMS 200	[02] BMS 200	-206.678	-167.818	-232.812	-5.11928
BMS 210	[00] BMS 210	-200.425	-166.787	-215.127	-3.76733
BMS 211	[00] BMS 211	-198.373	-166.546	-221.891	-5.44791
BMS 193	[00] BMS 193	-214.196	-166.093	-239.793	-2.32188
BMS 170	[01] BMS 170	-208.403	-164.463	-252.144	-1.06538
BMS 211	[01] BMS 211	-192.452	-164.362	-219.261	-2.10384
BMS 225	[00] BMS 225	-199.605	-163.26	-215.509	-3.68641
BMS 199	[00] BMS 199	-205.856	-163.117	-228.566	-0.42034

**Table 3:** Virtual screened drugs docking result with reference to high-affinity BMS- 202 against PubChem

Ligand	Filename	MolDock Score	Rerank Score	Interaction	HBond
127263272	[00]127263272	-193.261	-151.74	-225.408	-4.52206
126843234	[00]126843234	-178.19	-146.264	-199.562	-2.24356
127263272	[02]127263272	-180.754	-145.388	-211.309	-0.54806
126843234	[01]126843234	-174.74	-144.189	-199.801	-2.31062
126843234	[03]126843234	-169.764	-144.102	-199.117	0
126843234	[02]126843234	-176.053	-140.85	-199.719	-0.92555
127263272	[03]127263272	-176.215	-140.061	-195.578	-0.07669
127263272	[01]127263272	-177.071	-136.306	-201.379	-1.36358
127263272	[04]127263272	-167.671	-135.862	-200.165	-0.54703
126843234	[04]126843234	-164.291	-112.76	-185.444	-4.55516

**Table 4:** Virtual screened drugs docking result with reference to high-affinity BMS- 202 against Zinc

Ligand	Filename	MolDock Score	Rerank Score	Interaction	HBond
ZINC22037432_1	[00] ZINC22037432_1	-178.601	-143.428	-193.535	-2.5
ZINC01846354	[00] ZINC01846354	-179.375	-143.28	-180.818	-3.95799
ZINC00000101	[00] ZINC00000101	-178.673	-141.842	-179.504	-5.55762
ZINC22037436_1	[00] ZINC22037436_1	-169.848	-136.653	-185.571	-3.464
ZINC14684103	[00] ZINC14684103	-176.619	-135.147	-176.894	-0.08033
ZINC04652360	[00] ZINC04652360	-174.342	-134.726	-168.606	0
ZINC22037432_1	[03] ZINC22037432_1	-166.457	-134.52	-185.326	0
ZINC33844575	[02] ZINC33844575	-164.016	-134.485	-168.029	-0.49184
ZINC72266866	[01] ZINC72266866	-150.726	-133.76	-176.745	-0.13166
ZINC42479148	[00] ZINC42479148	-166.813	-132.496	-167.666	-7.91148

**Table:** Drug-Drug comparative study

Virtual Screened Drug CID: 127263272	Established drug CID:117951478
Energy overview: Descriptors	MolDock Score	Rerank Score	MolDock Score	Rerank Score
Total Energy	-193.236	-151.724	-200.215	-161.944
External Ligand interactions	-225.405	-188.661	-226.851	-191.146
Protein - Ligand interactions	-225.405	-188.661	-226.851	-191.146
Steric (by PLP)	-220.879	-151.523	-221.615	-152.028
Steric (by LJ12-6)		-33.554		-34.971
Hydrogen bonds	-4.526	-3.584	-5.236	-4.147
Internal Ligand interactions	32.169	36.937	26.636	29.202
Torsional strain	20.749	19.463	12.76	11.969
Torsional strain (sp2-sp2)		3.801		3.798
Hydrogen bonds		0		0
Steric (by PLP)	11.42	1.964	13.876	2.387
Steric (by LJ12-6)		11.71		11.048

**Table 6:** ADMET predicted profile (classification analysis)

PD1-PDL1 inhibitor Established drug (BMS-202) CID:117951478	XGIQBUNWFCCMAS-UHFFFAOYSA-N Virtual Screened Drug CID: 127263272
Model Absorption	Result	Probability	Probability
Blood-Brain Barrier	BBB+	0.6472	0.7091
Human Intestinal Absorption	HIA+	0.9776	0.9935
Caco-2 Permeability	Caco2+	0.5102	0.5251
P-glycoprotein Substrate	Substrate	0.862	0.8376
P-glycoprotein Inhibitor	Non-inhibitor	0.8351	0.8626
Non-inhibitor	0.9433	0.9246
Renal Organic Cation Transporter	Non-inhibitor	0.7057	0.7043
Distribution
Subcellular localization	Mitochondria	0.8594	0.812
Metabolism
CYP450 2C9 Substrate	Non-substrate	0.7944	0.7499
CYP450 2D6 Substrate	Non-substrate	0.6615	0.6547
CYP450 3A4 Substrate	Substrate	0.5915	0.6428
CYP450 1A2 Inhibitor	Inhibitor	0.5345	0.5766
CYP450 2C9 Inhibitor	Non-inhibitor	0.876	0.8296
CYP450 2D6 Inhibitor	Non-inhibitor	0.7646	0.716
CYP450 2C19 Inhibitor	Non-inhibitor	0.8901	0.8184
CYP450 3A4 Inhibitor	Non-inhibitor	0.6304	0.5114
CYP Inhibitory Promiscuity	Low CYP Inhibitory Promiscuity	0.8936	0.852
Excretion
Toxicity
Human Ether-a-go-go-Related Gene Inhibition	Weak inhibitor	0.9626	0.96
Inhibitor	0.8081	0.8063
AMES Toxicity	Non AMES toxic	0.7298	0.6513
Carcinogens	Non-carcinogens	0.8488	0.7883
Fish Toxicity	High FHMT	0.9277	0.9499
Tetrahymena Pyriformis Toxicity	High TPT	0.9196	0.9733
Honey Bee Toxicity	Low HBT	0.8083	0.8412
Biodegradation	Not ready biodegradable	0.9959	1
Acute Oral Toxicity	III	0.7035	0.6799
Carcinogenicity (Three-class)	Non-required	0.6788	0.6531

**Table 7:** ADMET Predicted Profile (regression analysis)

PD1-PDL1 inhibitor Established drug CID:117951478	XGIQBUNWFCCMAS-UHFFFAOYSA-N Virtual Screened Drug CID: 127263272
Model Absorption	Unit	Value	Value
Aqueous solubility	LogS	-2.3186	-2.7996
Caco-2 Permeability	LogPapp, cm/s	1.1475	1.212
Toxicity
Rat Acute Toxicity	LD50, mol/kg	2.4295	2.5248
Fish Toxicity	pLC50, mg/L	1.6327	1.4204
Tetrahymena Pyriformis Toxicity	pIGC50, ug/L	0.3436	0.4986

**Table 8:** Comparative ADMET profile of the test ligands and the control

Blood-Brain Barrier	Human Intestinal Absorption	AMES Toxicity	Carcinogenicity	LD50 in rats
PD1-PDL1 inhibitor BMS-202	0.6472	0.9776	0.7298	Non- carcinogenic	2.4295
XGIQBUNWFCCMAS-UHFFFAOYSA-N	0.7091	0.9935	0.6513	Non- carcinogenic	2.5248
BMS 198	0.7737	0.9901	0.6961	Non- carcinogenic	2.4876
BMS 200	0.7617	0.8816	0.7426	Non- carcinogenic	2.7858
126843234	0.8398	0.9692	0.6886	Non- carcinogenic	2.4431

**Table 9:** Cytotoxicity study result for the best virtual screened compound that is compound (PubChem CID 127263272)

Pa	Pi	Cell-line	Cell-line name	Tissue/organ
0.516	0.035	Kasumi 1	Childhood acute myeloid leukemia with maturation	Haematopoietic and lymphoid tissue
0.334	0.048	CCRF-CEM	Childhood T acute lymphoblastic leukemia	Blood
0.256	0.07	NCI-H69	Small cell lung carcinoma	Lung
0.301	0.139	U-266	Plasma cell myeloma	Blood
0.134	0.043	D54	Glioblastoma	Brain
0.103	0.02	MOLT-3	T-lymphoblastic leukemia	Blood
0.23	0.171	Hs-578T	Invasive ductal breast carcinoma	Breast
0.125	0.094	A2780cisR	Cisplatin-resistant ovarian carcinoma	Ovarium
0.155	0.136	Ramos	Burkittslymhoma B-cells	Blood
0.289	0.28	NALM-6	Adult B acute lymphoblastic leukemia	Haematopoietic and lymphoid tissue
